# A retrospective review of a large series of groin hernia patients operated with robotically assisted laparoscopic technique (R-TAPP)

**DOI:** 10.1007/s11701-022-01474-x

**Published:** 2022-10-25

**Authors:** Johan Bondi, Hans Gunnar Botnen, Oliver Baekkelund, Sigrid Groven

**Affiliations:** Department of Surgery, Drammen Hospital, Vestre Viken Hospital Trust, Drammen, Norway

**Keywords:** Groin hernia, Robotic, R-TAPP, Follow-up

## Abstract

We have reviewed the patient outcome and the feasibility of robotically assisted inguinal hernia repair (R-TAPP) from the first 4-years period after its introduction in our department in a Scandinavian Public Health hospital. A total of 226 hernia repairs were performed in 195 patients (31 bilateral hernias). 160 patients had primary hernias, whereas 35 had recurrent hernias. Of the recurrent hernias, three had recurred twice. The majority of the hernias were in the right groin (53.3%) and the lateral location was the most common (65.0%). The hernia was scrotal in 29 cases. The mean operation time was significantly reduced throughout the observation period for our cohort, i.e. from 81 to 57 min (*p* < 0.001). The operation time was 27 min faster (mean value) in unilateral vs bilateral hernias and 19 min faster (mean value) in primary vs residual hernias. There were no statistically significant differences in operation time between lateral and medial hernias, and no differences in operation time between the obese and normal-weight cases. We experienced four severe per-operative complications (4/226; 1.8%): two cases of abdominal wall bleeding subsequently undergoing intravascular coiling, one perforation of the urinary bladder and one perforation of small bowel that were both closed by direct suture intraoperatively. There were no conversions to laparoscopy or open procedure. One hernia recurred during the observation period. Our findings suggest that the R-TAPP procedure in a Scandinavian Public Health hospital’s surgical department is both safe and feasible.

## Introduction

Hernia repair is one of the most frequent procedures in general surgery in both men and women [1]. Compared to the open technique, the laparoscopic approach has shown to imply a lower incidence of wound infections, fewer postoperative hematomas, less chronic pain and a shorter time to normal daily activity [2]. The first robotically assisted laparoscopic inguinal hernia repair was reported in 2007, as a supplementary procedure to a prostatectomy [3]. Since then, several reports have been published on robotic hernia repairs, but often on a limited number of patients [4–11]. In addition, multicenter studies [12, 13] and single surgeon experiences are published [14, 15].

Today, robotically assisted surgery for hernias per se is a common method worldwide. In the USA the use of robotic surgery for inguinal hernia repair increased from under 1 percent to nearly 30 percent from 2012 through 2018 [16]. Accordingly, the *da Vinci* robot (Intuitive Surgical, Sunnyvale, CA, USA) is now an increasingly common tool for minimally invasive surgery in many institutions, due to its properties facilitating operating in small spaces through increased handiness and a steady 3D image.

The best and most effective use of a robotic system in the day-to-day clinical setting of a large, busy surgical department is still to be fully established.

We have reviewed the patient outcome and the feasibility of robotically assisted inguinal hernia repair (R-TAPP) from the first 4-years period after its introduction in our department in a Scandinavian Public Health hospital.

## Materials and methods

### Patients, data collection, definitions and analysis

We have reviewed all patients operated with the R-TAPP technique in our hospital in the period between 1.1.2018 and 31.12.2021. The patients were retrospectively identified through our hospital’s digital patient medical record system (DIPS, Bodø, Norway) and subsequently included in our study database in which the following types of parameters were added: patient characteristics, hernia characteristics, details of the surgical procedure, intra- and postoperative complications and short term recurrence rates.

In our institution, serving about 220,000 people and providing the full repertoire of colorectal and abdominal wall surgery, the straight-stick laparoscopic TEP performed in the outpatient setting, has been the standard for groin hernia repair. An open approach is offered patients unfit for general anesthesia or in whom the laparoscopic technique is considered unsuitable. Thus, the patients offered a robotically assisted repair therefore consists in this material mainly of patients in an expected need of an over-night stay in the hospital due to their medical situation or by practical considerations.

All patients were encouraged to contact our department in case of complications or complaints after their hernia repair and our hospital serves an area in Norway with a low degree of migration and no other surgical centers offering hernia repairs. Hence, no re-contact with our department is in this review considered as having no clinically relevant problems postoperatively.

Operation time is defined from the opening of the skin for the introduction of the first trocar until the finishing of skin-closure after the procedure.

Hernia size is not accurately defined, but is based on the surgeon’s subjective opinion.

The study was approved by the Patient Safety Representative in the Vestre Viken Health Region, Norway.

The data were analyzed using SPSS v 26 (IBM, Armonk, NY, USA). Comparison of means were performed by Student’s *t* test for independent samples. Significance was set at *p* < 0.05.

### Surgical procedure

Six different consultant general surgeons performed the R-TAPPs. All six surgeons were experienced in hernia surgery and laparoscopy prior to the implementation of robotically assisted procedures in our institution. The R-TAPP was chosen as one of the procedures suitable for start-up of robotic surgery in our department, and all surgeons underwent the obligatory technical training according to the regulations of the provider of the robot. In addition, we visited experienced robotic surgeons C Balecer (Phoenix, AZ, USA) and F Muysoms (Gent, Belgium) to learn the R-TAPP procedure prior to implementing the procedure in our hospital.

All procedures were performed using the *da Vinci* Xi robot, with a standard set-up using three trocars on a horizontal line through the upper abdominal wall. The lower half of the peritoneal cavity was briefly overviewed before the hernia was addressed. The peritoneum was incised horizontally from just cranial to the anterior superior iliac spine (ASIS), to the midline (or to the contralateral ASIS in bilateral hernias). Then the peritoneum and hernia sac, with its adjacent lipoma if present, were reduced. In all procedures the hernia opening was covered by a self-fixating polypropylene mesh (ProGrip™, Medtronic, Dublin, Ireland), before covering the mesh with the peritoneal flap by a continuous barbed monofilament 3-0 suture (V-Loc™, Medtronic, Dublin, Ireland). Finally, the trocar openings were closed intracutaneously (Monosyn® Quick 4-0, B. Braun, Melsingen, Germany).

All patients were offered to stay overnight in the hospital, but could be discharged the same day if there were no signs of early complications after a few hours of in-hospital observation.

## Results

### Patient characteristics

We performed hernia repairs in 195 patients (29 women, 166 men), distributed as follows: 29 in 2018, 63 in 2019, 44 in 2020 and 59 in 2021. The median observation time was 24.1 months (range 2.5–49.6 months).

The mean age was 66 years (range 25–89 years). The majority of the patients had a generally good health; 77% (*n* = 150) had an ASA score ≤ 2, for details see Table 1. The mean patient BMI was 25 (range 18–37), of whom 48% (*n* = 94) were overweight (Table 1).

**Table 1:** Patients characteristics

	Age [years]	ASA score*	BMI^†^
Mean	66	2	25
Median	67	2	25
Minimum	25	1	18
Maximum	89	4	37

*American Society of Anesthesiologist-score for assessing fitness of patients before surgery (grade I = normal physical health, grade II = mild systemic disease, grade III = severe systemic disease, grade IV = severe systemic disease that is a constant threat to life)

^†^Body Mass Index = kg/m^2^. BMI ≥ 25 indicates overweight

### Hernia characteristics

In this cohort of 195 patients, 31 had bilateral hernias, so that 226 hernia repairs were performed. In eleven of the patients with a lateral hernia, an additional small medial hernia was also observed intraoperatively, and in four other patients a small femoral hernia was found *en passant*. In two patients, an obturator hernia was found in addition to an already diagnosed femoral and inguinal hernia, respectively. All incidentally detected hernias were smaller than the hernia constituting the indication for the operation. Taken together a total of 243 hernias were repaired in our material.

All patients had symptomatic hernias. Seven patients had experienced near incarceration episodes prior to surgery, but neither was any of the patients operated in an acute setting, nor for an incarcerated/non-reducable hernia.

160 patients had primary hernias, whereas 35 had recurrent hernias. Of the recurrent hernias, 3 had recurred twice. The majority of the hernias were in the right groin (53.3%) and the lateral location was the most common (158/243; 65.0%), see Table 2.

**Table 2:** Hernia characteristics

	*n*	%
Previous operations		
0	160	70.0
1	32	14.1
2	3	1.3
Side*		
Right groin	104	53.3
Left groin	60	30.8
Bilateral	31	15.9

Location			
	Preoperatively detected	Intraoperative additional detection	*n* sum
	*n*	*n*	
Lateral	158	0	158
Medial	63	11	74
Femoral	5	4	9
Obturator canal	0	2	2
*n* sum	226	17	243

Forty-seven of the hernias were described as large (47/243; 19.3%), 21 out of 243 (8.6%) as small and the reminder as intermediate size or not reported. Twenty-nine (29/243; 11.9%) were scrotal hernias.

In 38 out of 243 (15.6%) hernias the sack was occupied by small or large intestine, whereas in 47 (19.3%) the sack was filled with fat/lipoma. In two patients (2/195; 1.0%) a small tab of the urinary bladder protruded into the hernia sack. In most cases (104/195; 53.3%) the sack was either empty or its content not reported.

In the majority of the cases (171/195; 87.7%), no pre-operative radiological diagnostics were performed. An ultrasound examination of the groin area was performed in 9 patients (4.6%), and a CT scan was used to verify a hernia in 15 patients (7.7%).

All hernia openings were covered by a self-fixating polypropylene mesh. In 81 of 226 (35.8%) herniaplasties a mesh sized 16 × 12 cm was used, whereas in 145 of the 226 procedures (63.9%) a preformed mesh sized 15 × 10 cm was applied. One patient only got a cross-stich closing of the inner opening of a lateral hernia with a multifilament suture, because an intraoperative transmural lesion of small bowel was discovered, and an application of a synthetic mesh in a potentially contaminated space was considered hazardous.

### Intraoperative characteristics

**Fig. 1:**
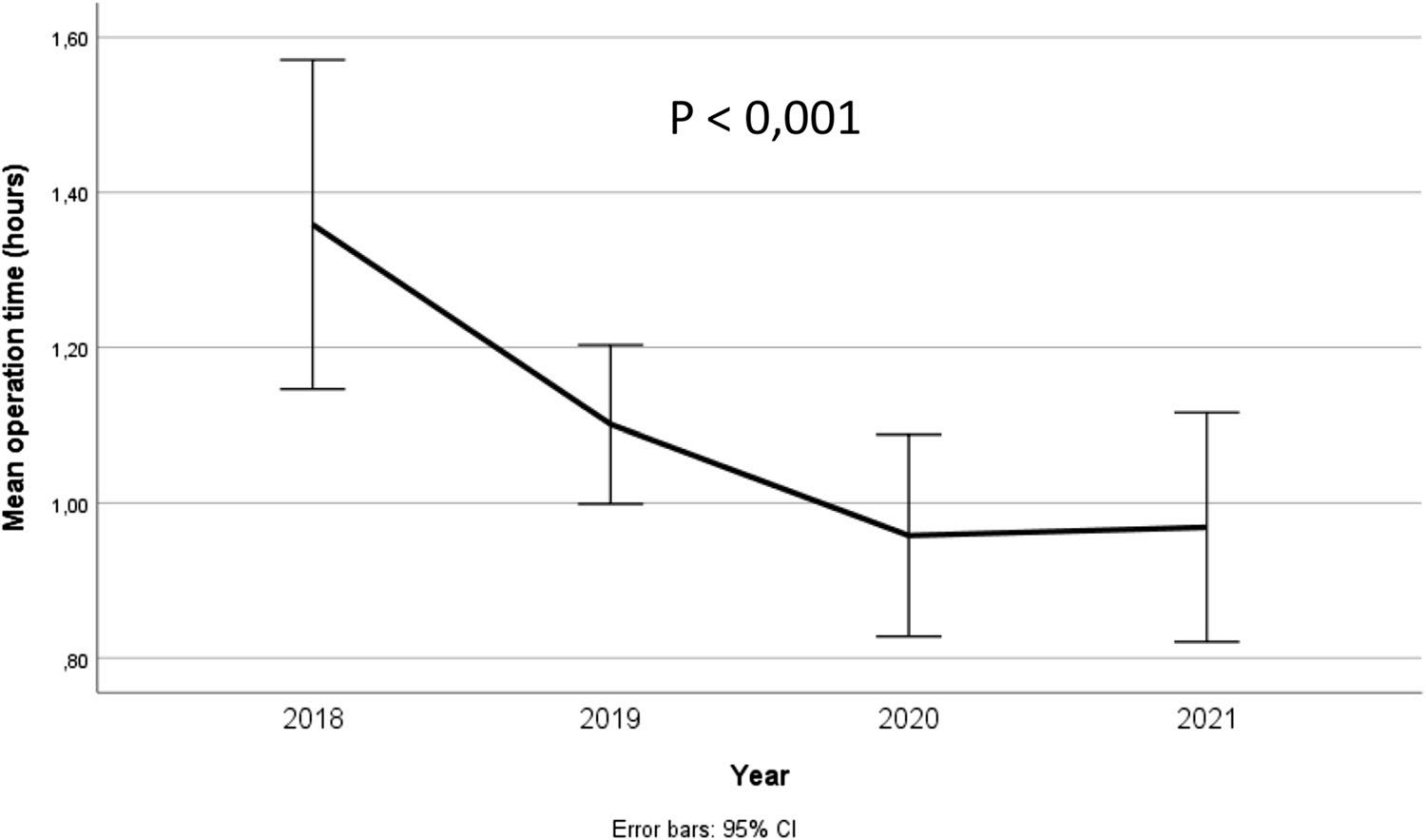
Mean operation time during the observation period

The mean operation time was significantly reduced throughout the observation period for our cohort, i.e. from 81 to 57 min (*p* < 0.001), Fig. 1.

The two most long-lasting operations were performed during the first year. The longest operation was performed on a recurrent hernia with difficult dissection due to previous prostate surgery obliterating the anatomical layers and included comprehensive adhesiolysis.

The operation time was 27 min faster (mean value) in unilateral vs bilateral hernias and 19 min faster (mean value) in primary vs residual hernias. There were no statistically significant differences in operation time between lateral and medial hernias, and no differences in operation time between the obese and normal-weight cases (Table 3).

**Table 3:** Operation times (hours:minutes)

	All	Primary	Recurrent	Bilateral	Primary unilateral
Mean	1:07	1:04	1:20	1:29	0:57
Median	1:13	1:09	1:28	1:30	1:06
Shortest	0:28	0:28	0:34	0:50	0:28
Longest	3:11	2:31	3:11	2:31	2:20

Comparison of operating times			
	Difference (minutes)	*p* value	95% CI
Primary vs residual hernias	19	0.025	0.02 to 0.36
Unlateral vs bilateral hernias	27	0.003	0.09 to 0.44
Medial vs lateral hernias	10	0.19	− 0.25 to 0.05
Normalweight vs obese	2	0.68	0.95 to 1.16

We experienced four severe peroperative complications (4/226 = 1.8%): Two cases of abdominal wall bleeding that was treated by radiologically assisted intravascular coiling, one perforation of the urinary bladder that was closed by direct suture, and one perforation of small bowel that altered the initial plan for the procedure (as described under *Hernia characteristics*).

We had no conversions to laparoscopy or open repair.

### Postoperative characteristics

During the postoperative observation time, we found only one patient with a recurrence of the R-TAPP. The relapse was evident after 27 months and was repaired with a second R-TAPP one month later. The original mesh was found to be dislocated cranially, no longer covering the hernia opening. A new mesh was applied.

The most common complication was the development of a seroma/hematoma. This was observed in 16 patients (8.2%). Two of these patients (2/16) used an anticoagulant drug daily.

The most severe complications were the two cases of abdominal wall hemorrhage, which continued to bleed postoperatively and required radiologically assisted coiling. One of these patients also developed an acute myocardial infarction following this incident. They both had huge hernias, one of which protruded to the scrotum. One of the patients took warfarin (INR 2.1 on the day of surgery) because of a mechanical aortic valve and the other took acetylsalicylic acid daily as primary prophylaxis in hypertension. The postoperative complications are listed in full in Table 4.

**Table 4:** Postoperative complications

Complication type	*n*	%	Consequence	Clavien-Dindo score
Hernia recurrence	1	0.4	Re-operated with R-TAPP after 3 years	3b
Chronic pain [> 3 months]	4	1.8	2 have received steroid-injections for this	2
Seroma/hematoma	16	7.0	3 evacuated by needle, 1 surgically removed	1 og 3b
Bleeding	2	0.9	Coiling	3/4
Urinary retention	1	0.4	Urinary catheter for 6 weeks [Then a trans-urethral resection in an elective setting]	2
Bowel obstruction	1	0.4	Reoprated laparoscopically, reduced small bowel trapped in the peritoneal pocket made during R-TAPP	3b

Four patients were re-admitted within the first 30 days after the operation, of whom only one patient needed surgery. He suffered from an acute small bowel obstruction due to a bowel entrapment in a defect in the peritoneal flap from the R-TAPP which was laparoscopically reduced. One patient received needle aspitatin of a seroma, one patient presented a seroma which did not require any treatment and one patient stayed overnight due to postoperative pain.

Neither superficial nor deep infections were reported during the observed postoperative time.

## Discussion

To our knowledge, this is the only review of R-TAPP from a Scandinavian public health institution and among the larger series reported from a single center [4–11].

Our results support most previous reports demonstrating robotically assisted groin hernia repair to be both safe and feasible (se [17] for a review).

The overall complication rate (Clavien-Dindo score ≥ 2) of 11% is comparable to the findings of others [4–11]. We found one hernia relapse in our series of 227 repairs, even though the follow-up time is relatively short (mean 24 months).

It has been argued that the robotic approach may require longer operation time, at least in the unilateral cases [17]. The operating time in our material is in the lower range compared to previous reports and declined through the observation period (Fig. 1). In a recent report by Proietti et al. [18], the learning curve for R-TAPP was addressed, and they found that 43 procedures were needed for the surgeon to obtain the required skills and a significantly reduced operation time. In our review, three of the six surgeons obtained this number of operations during the observation time. Further, we found no difference in the operation time between normal-weight and over-weight patients. This is in compliance to previous findings [19].

In our institution the straight-stick laparoscopic TEP performed in the outpatient setting has been the standard for groin hernia repair. An open approach is offered patients unfit for general anesthesia or for whom the laparoscopic technique is considered unsuitable. The patients offered a robotically assisted repair therefore consists in this material mainly of patients in an expected need of an over-night stay in the hospital due to co-morbidity or by practical considerations, or if a time-consuming procedure is expected, e.g. surgery for a recurrent hernia. Thus, it could be argued that potential bias in our findings is at least not due to less complex cases or a selection of the patients assumed best fitted for the R-TAPP procedure.

Muysoms et al. [20] found robot-assisted laparoscopic inguinal repair to be more expensive than conventional laparoscopy. In their review, more patients were treated as outpatients in the robotic group and the postoperative complications were infrequent and mild. We believe that by taking the appropriate measures, the R-TAPPs can be performed routinely in the outpatient setting as well, reducing the cost accordingly.

During the observed period in this study, four of the six surgeons have also started to do 4-arm robotic colorectal resections, rectopexies and complex ventral hernias including component separation. We believe the experience achieved through the 3-arm less complex procedure of R-TAPP, the transition to the more comprehensive procedures have been both smooth and safe. Therefore, in our opinion the R-TAPP also serves as a fairly simple and safe procedure to introduce to robotic surgeons before taking on more advanced cases.

However, the robot requires a relatively large operating room and can imply a large economical one-time investment for a clinical department. The robot system is often shared between different departments performing robotic surgery, to reduce cost and optimize the active time for the system. In our institution 557 groin hernias were repaired by ordinary laparoscopy and 128 by open technique during this review’s observation time. Hence, we believe that even in a busy surgical department as we represent, the inclusion of R-TAPP has been both feasible and advisable.

## Conclusion

We found in our retrospective review that the introduction of the R-TAPP procedure in a Scandinavian Public Health hospital’s surgical department is both safe and feasible.
